# Reproducibility of a Self-administered Food Frequency Questionnaire Used in the 5-year Follow-up Survey of the JPHC Study Cohort I to Assess Food and Nutrient Intake

**DOI:** 10.2188/jea.13.1sup_115

**Published:** 2007-11-30

**Authors:** Satoshi Sasaki, Junko Ishihara, Shoichiro Tsugane

**Affiliations:** 1 Epidemiology and Biostatistics Division, National Cancer Center Research Institute East.; 2 Cancer Information and Epidemiology Division, National Cancer Center Research Institute.

**Keywords:** reproducibility, nutrient, food group, food frequency questionnaire

## Abstract

We examined the reproducibility of a self-administered semiquantitative food frequency questionnaire (FFQ) used in the 5-year follow-up survey for the Japan Public Health Center-based prospective Study on cancer and cardiovascular diseases (JPHC Study) to estimate nutrient and food intake by using repeated FFQs at a 1-year interval in 101 men and 108 women. Between energy and each of 32 nutrients, the correlation coefficients in crude values varied from 0.41 for vitamin B_12_ to 0.83 for alcohol (median=0.59) in men and 0.52 for alpha-carotene to 0.77 for iron (median=0.67) in women. In 21 food groups, it varied from 0.42 for seasonings and spices to 0.80 for pickled vegetables (median=0.61) in men and 0.45 for seasonings and spices and 0.74 for pulses, milks, and pickled vegetables (median=0.63) in women. The correlation coefficients for the energy-adjusted values (medians were 0.49 and 0.50 for nutrients and 0.50 and 0.49 for food groups in men and women, respectively) were somewhat lower than for the crude values. The difference in mean intakes between the two FFQs was less than 10% in most of the nutrient and food groups. The results suggest that the reproducibility of the FFQ used for the JPHC study was moderate to high in most of the nutrient and food groups.

Nutrient and food intakes have been assessed with a self-administered food frequency questionnaire (FFQ) in several nutritional epidemiologic studies for chronic diseases.^1^^,^^2^ In most observational epidemiologic studies, single measurements of dietary habits have been used as representative of long-term dietary habits of individuals. To use a questionnaire for this purpose, the reproducibility as well as the validity should be examined to assure the consistency of the dietary habits in the target population. We therefore examined reproducibility of a FFQ used in the 5-year follow-up survey of the Japan Public Health Center-based prospective Study on cancer and cardiovascular diseases (JPHC Study) using two data sets obtained one year apart.

## METHODS

The study design and subject characteristics have been reported elsewhere.^3^ Subjects included in the analysis were 101 men and 108 women who completed FFQ twice at a one-year interval in the Ninohe, Yokote, Saku and Ishikawa Public Health Center (PHC) areas in the JPHC Study. In the Ninohe, Yokote and Saku PHC areas, the first FFQ (FFQ1) was conducted in February, 1995 as a part of the 5-year follow-up survey for the entire JPHC study cohort I, and the second FFQ (FFQ2) was conducted in February, 1996. In the Ishikawa PHC area, the FFQ1 was conducted in February, 1995, and the FFQ2 in February, 1996. In the previous report on the design of this study,^3^ the FFQ immediately after the completion of dietary records (DR) was described as the FFQ for validity (FFQ_V) and the other as the FFQ for reproducibility (FFQ_R) in order to distinguish the validity of the FFQ in conjunction with DR. For the purpose of this report, however, we aimed to examine the reproducibility of two FFQs at a one-year interval in chronological order. Therefore, for the Ninohe, Yokote and Saku PHC areas, the FFQ_V was regarded as FFQ1, and the FFQ_R as FFQ2. For Ishikawa PHC, the FFQ_R was regarded as FFQ1 and the FFQ_V as FFQ2.

The methods for computing nutrient and food intakes from the FFQ have been described in this Supplement.^4^ The mean crude intakes of energy, 32 nutrients and 21 food groups were calculated from each FFQ (the first and the second FFQs). Vegetables were divided into three groups, i.e., green and yellow, non-green and yellow (namely, other), and pickled vegetables. Beverages were divided into two groups, alcoholic and non-alcoholic. Intake of sugars and sweeteners was not computed in this FFQ so the food group was not included in the analysis.

Mean energy-density values were calculated as intake of nutrient and food groups per 1000 kcal. The percent difference between the first and second FFQ was computed by dividing the difference in mean intake between the two FFQs by the FFQ1 mean: (FFQ2 mean-FFQ1 mean)/FFQ1 mean.

Intakes of nutrients and foods were adjusted for total energy intake using a residual model.^5^ Spearman rank correlation coefficients between the two FFQs were computed for crude and energy-adjusted intakes. The Spearman rank correlation coefficient was used for the correlation analysis because the distribution was skewed in most values. Because our purpose was to quantify measurement error rather than test a hypothesis, p values were not presented for correlation coefficients. All the analyses were performed separately for men and women. The computation was performed using the data with the 4 areas combined.

## RESULTS

Table 1 shows the mean and standard deviation (SD) of nutrient intakes in crude values and the differences between the two FFQs. The percent difference was less than 5% in most nutrients except for n-6 polyunsaturated fatty acid, sodium, carotenes (alpha- and beta-), vitamin B_12_, daidzein and genistein in men, and saturated fatty acid, n-6 polyunsaturated fatty acid, alcohol, alpha-carotene, vitamin B_12_ and genistein in women.

**Table 1.** Means and standard deviations of energy and nutrient intakes (crude values) assessed with two FFQs administered at one-year interval

Intake	Men (n=101)											Women (n=108)										
	
	FFQ1^1^				FFQ2^2^				Difference	p-value^3^	%^4^ difference	FFQ1^1^				FFQ2^2^				Difference	p-value^3^	%^4^ difference
			
	Mean	±	SD	Median	Mean	±	SD	Median				Mean	±	SD	Median	Mean	±	SD	Median			
Energy (kcal/day)	2305	±	691	2272	2323	±	706	2279	18	0.791	1	1983	±	858	1819	2030	±	600	1892	47	0.522	2
Protein (g/day)	86.2	±	35.6	77.7	87.0	±	35.7	83.6	0.8	0.788	1	80.3	±	46.3	71.1	81.8	±	30.5	75.1	1.5	0.717	2
Total fat (g/day)	63.4	±	27.7	59.8	63.9	±	28.4	60.2	0.6	0.837	1	62.5	±	37.3	54.3	65.0	±	28.8	56.8	2.5	0.465	4
SFA^5^ (g/day)	18.2	±	9.0	17.0	18.2	±	8.4	17.0	0.1	0.936	0	17.7	±	9.1	15.5	18.8	±	9.7	15.7	1.1	0.283	6
MUFA^6^ (g/day)	23.8	±	10.7	22.9	23.8	±	11.4	22.0	0.0	0.989	0	23.3	±	14.3	19.8	24.2	±	11.1	21.5	0.8	0.531	4
PUFA^7^ (g/day)	14.6	±	6.5	13.2	15.1	±	7.0	14.2	0.5	0.429	3	14.8	±	10.6	12.3	15.2	±	6.6	13.9	0.5	0.581	3
n-3 PUFA^7^ (g/day)	3.7	±	2.1	3.3	3.6	±	2.0	3.4	-0.1	0.772	-2	3.8	±	3.2	3.0	3.6	±	1.7	3.2	-0.1	0.650	-3
n-6 PUFA^7^ (g/day)	10.9	±	4.6	10.0	11.4	±	5.2	10.8	0.5	0.235	5	11.0	±	7.5	9.4	11.6	±	5.1	10.5	0.6	0.334	6
Carbohydrate (g/day)	303.3	±	100.2	290.7	306.1	±	98.1	287.3	2.8	0.758	1	272.7	±	97.4	259.7	278.6	±	73.4	265.0	6.0	0.459	2
Alcohol (g/day)	23.5	±	23.1	22.7	23.3	±	25.5	17.8	-0.2	0.922	-1	1.5	±	7.3	0.0	0.6	±	2.6	0.0	-0.9	0.119	-60
Calcium (mg/day)	652.7	±	393.0	590.7	662.4	±	333.3	614.6	9.7	0.800	1	671.7	±	400.1	581.3	690.7	±	308.9	645.9	19.1	0.612	3
Phosphorus (mg/day)	1372	±	551	1327	1382	±	525	1342	10	0.832	1	1283	±	649	1141	1314	±	453	1221	31	0.586	2
Iron (mg/day)	11.7	±	5.1	11.0	11.8	±	4.9	11.7	0.1	0.798	1	11.7	±	7.2	10.5	1.9	±	4.6	10.9	0.2	0.785	1
Sodium (mg/day)	5603	±	2618	5311	5898	±	3095	5611	295	0.236	5	5315	±	3170	4720	5302	±	2296	4974	-13	0.959	0
Potassium (mg/day)	3196	±	1482	2961	3176	±	1232	3077	-20	0.876	-1	3249	±	1882	2799	3272	±	1111	3130	22	0.884	1
Retinol (*µ*g/day)	620.5	±	568.3	511.1	613.7	±	699.2	415.5	-6.8	0.922	-1	598.4	±	711.1	417.7	590.6	±	603.7	456.5	-7.8	0.916	-1
Carotene (*µ*g/day)	3804	±	3139	3308	3356	±	2244	2734	-448	0.130	-12	4043	±	3046	3291	4223	±	2918	3308	180	0.577	4
Alpha-carotene (*µ*g/day)	558.3	±	546.9	393.2	471.8	±	466.1	375.6	-86.5	0.118	-15	572.0	±	500.2	407.1	625.9	±	547.9	412.8	53.9	0.334	9
Beta-carotene (*µ*g/day)	3039	±	2594	2552	2689	±	1777	2291	-350	0.148	-12	3242	±	2504	2749	3378	±	2370	2667	136	0.612	4
Vitamin B_1_ (mg/day)	1.2	±	0.5	1.1	1.2	±	0.5	1.2	0.0	0.899	0	1.2	±	0.6	1.0	1.2	±	0.5	1.1	0.0	0.664	2
Vitamin B_2_ (mg/day)	1.7	±	0.8	1.6	1.7	±	0.7	1.6	0.0	0.794	-1	1.7	±	0.9	1.5	1.7	±	0.6	1.6	0.0	0.937	0
Niacin (mg/day)	20.3	±	8.2	18.5	19.6	±	7.9	18.4	-0.8	0.296	-4	17.9	±	11.1	15.5	17.8	±	6.5	16.1	-0.1	0.907	-1
Vitamin C (mg/day)	166.3	±	118.6	157.1	163.1	±	89.7	147.4	-3.2	0.731	-2	191.4	±	161.5	155.7	188.6	±	88.7	174.0	-2.8	0.832	-1
Cholesterol (mg/day)	334.2	±	156.0	317.7	333.8	±	175.5	309.7	-0.4	0.978	0	319.0	±	170.4	307.4	328.9	±	195.7	297.6	10.0	0.571	3
Vitamin B_6_ (mg/day)	2.0	±	0.8	1.8	1.9	±	0.8	1.8	-0.1	0.423	-3	1.7	±	1.1	1.5	1.7	±	0.6	1.6	0.0	0.902	-1
Vitamin B_12_ (*µ*g/day)	12.0	±	8.4	9.8	11.3	±	8.1	10.4	-0.7	0.326	-6	11.2	±	10.9	8.7	103	±	6.4	8.6	-0.8	0.393	-7
Folate (*µ*g/day)	318.2	±	152.7	286.8	312.7	±	131.2	293.2	-5.5	0.695	-2	324.9	±	189.4	274.9	326.2	±	127.7	303.8	1.3	0.939	0
Selenium (*µ*g/day)	126.1	±	58.1	111.4	125.2	±	62.7	117.7	-0.9	0.873	-1	116.3	±	70.3	96.2	117.4	±	50.6	106.4	1.2	0.850	1
Total dietary fiber (g/day)	14.5	±	7.5	13.4	14.8	±	6.6	14.1	0.3	0.580	2	15.9	±	10.3	13.9	16.2	±	6.2	15.4	0.2	0.763	2
Water-soluble fiber (g/day)	2.3	±	1.5	2.0	2.4	±	1.4	2.0	0.1	0.364	4	2.7	±	2.0	2.1	2.8	±	1.3	2.6	0.1	0.711	2
Water-insoluble fiber (g/day)	10.2	±	5.4	9.4	10.3	±	4.5	9.8	0.2	0.704	1	11.2	±	7.5	9.5	11.3	±	4.4	10.8	0.1	0.897	1
Daidzein (mg/day)	18.1	±	12.1	15.4	19.6	±	14.7	17.6	1.5	0.196	8	17.9	±	13.6	14.6	18.6	±	10.9	16.7	0.7	0.443	4
Genistein (mg/day)	30.7	±	20.6	25.9	33.5	±	25.2	29.0	2.7	0.173	9	30.8	±	25.8	24.1	32.5	±	20.3	28.5	1.8	0.343	6

^1^Food frequency questionnaire administered in February, 1995. ^2^Food frequency questionnaire administered in February, 1996

^3^Differences between FFQ1 and FFQ2 were tested by paired t-test. ^4^((FFQ2 mean - FFQ1 mean)/FFQ1 mean)

^5^Saturated fatty acid. ^6^Monounsaturated fatty acid. ^7^Polyunsaturated fatty acid.

Table 2 shows the mean and SD of nutrient intakes in energy-density values and the differences between the two FFQs. The percent difference was less than 5% in most nutrients except alcohol, carotenes (total, alpha- and beta-) in both sexes, and vitamin B_12_, daidzein and genistein in men.

**Table 2.** Means and standard deviations of nutrient intakes (energy-density values) assessed with two FFQs administered at one-year interval

Intake	Men (n=101)											Women (n=108)										
	
	FFQ1^1^				FFQ2^2^				Difference	p-value^3^	%^4^ difference	FFQ1^1^				FFQ2^2^				Difference	p-value^3^	%^4^ difference
			
	Mean	±	SD	Median	Mean	±	SD	Median				Mean	±	SD	Median	Mean	±	SD	Median			
Protein (g/day)	14.6	±	2.5	14.8	14.7	±	2.9	14.5	0.1	0.723	1	15.8	±	2.2	15.6	15.9	±	2.0	15.8	0.1	0.727	0
Total fat (g/day)	24.4	±	6.5	24.5	24.4	±	6.8	23.8	0.0	0.954	0	27.6	±	5.3	27.0	28.3	±	5.6	27.9	0.7	0.152	2
SFA^5^ (g/1000 kcal)	7.0	±	2.2	6.9	7.0	±	2.4	6.7	0.0	0.932	0	8.0	±	2.0	7.7	8.1	±	2.4	7.7	0.2	0.440	2
MUFA^6^ (g/1000 kcal)	9.2	±	2.9	9.0	9.1	±	2.9	8.7	-0.1	0.605	-2	10.3	±	2.3	10.0	10.5	±	2.4	10.1	0.2	0.232	2
PUFA^7^ (g/1000 kcal)	5.6	±	1.4	5.6	5.7	±	1.6	5.7	0.1	0.384	2	6.4	±	1.4	6.2	6.6	±	1.4	6.4	0.2	0.042	4
n-3 PUFA^7^ (g/1000 kcal)	1.4	±	0.5	1.4	1.4	±	0.5	1.4	0.0	0.482	-2	1.6	±	0.5	1.5	1.6	±	0.4	1.5	0.0	0.728	-1
n-6 PUFA^7^ (g/1000 kcal)	4.2	±	1.0	4.2	4.3	±	1.2	4.3	0.1	0.146	4	4.8	±	1.0	4.7	5.1	±	1.0	5.0	0.3	0.004	5
Carbohydrate (g/1000 kcal)	52.8	±	8.0	53.4	53.3	±	8.7	52.8	0.5	0.535	1	56.1	±	6.7	56.9	55.7	±	7.1	56.2	-0.4	0.541	-1
Alcohol (g/1000 kcal)	7.6	±	7.7	6.5	7.0	±	7.7	5.0	-0.5	0.305	-7	0.5	±	2.5	0.0	0.2	±	1.0	0.0	-0.3	0.167	-57
Calcium (mg/1000 kcal)	270.9	±	94.2	256.8	278.9	±	108.7	275.6	7.9	0.451	3	332.5	±	90.9	317.4	336.6	±	104.9	321.1	4.2	0.654	1
Phosphorus (mg/1000 kcal)	582.7	±	84.6	582.2	588.9	±	106.4	577.2	6.2	0.487	1	640.5	±	79.6	635.0	643.1	±	83.6	634.3	2.6	0.734	0
Iron (mg/1000 kcal)	5.0	±	1.1	4.9	5.0	±	1.2	4.9	0.0	0.799	0	5.7	±	1.1	5.6	5.8	±	1.2	5.6	0.0	0.621	1
Sodium (mg/1000 kcal)	2358	±	677	2349	2463	±	848	2385	104	0.136	4	2611	±	757	2622	2597	±	783	2580	-13	0.822	-1
Potassium (mg/1000 kcal)	1356	±	311	1344	1356	±	310	1339	-1	0.981	0	1605	±	324	1575	1617	±	316	1561	12	0.686	1
Retinol (*µ*g/1000 kcal)	260.8	±	195.9	227.0	248.9	±	212.4	190.9	-11.9	0.604	-5	279.4	±	254.6	212.6	275.8	±	212.2	259.1	-3.5	0.886	-1
Carotene (*µ*g/1000 kcal)	1609	±	952	1372	1444	±	859	1308	-164	0.073	-10	2020	±	998	1883	2136	±	1415	1731	117	0.369	6
Alpha-carotene (*µ*g/1000 kcal)	239.6	±	191.3	179.7	207.9	±	196.5	166.7	-31.7	0.086	-13	291.8	±	208.6	233.2	321.9	±	293.6	226.1	30.2	0.253	10
Beta-carotene (*µ*g/1000 kcal)	1282	±	777	1129	1154	±	668	1040	-128	0.081	-10	1615	±	812	1490	1705	±	1125	1371	90	0.391	6
Vitamin B_1_ (mg/1000 kcal)	0.5	±	0.1	0.5	0.5	±	0.1	0.5	0.0	0.842	0	0.6	±	0.1	0.6	0.6	±	0.1	0.6	0.0	0.654	1
Vitamin B_2_ (mg/1000 kcal)	0.7	±	0.2	0.8	0.7	±	0.2	0.7	0.0	0.671	-1	0.8	±	0.2	0.8	0.8	±	0.2	0.8	0.0	0.395	-2
Niacin (mg/1000 kcal)	8.7	±	1.7	8.9	8.4	±	1.8	8.4	-0.4	0.057	-4	8.8	±	1.6	8.4	8.7	±	1.5	8.6	-0.1	0.727	-1
Vitamin C (mg/1000 kcal)	70.9	±	32.6	70.0	69.5	±	30.7	70.6	-1.3	0.607	-2	92.7	±	41.1	85.2	94.0	±	35.9	89.9	1.4	0.709	1
Cholesterol (mg/1000 kcal)	142.9	±	47.0	144.8	140.9	±	54.9	138.1	-2.0	0.692	-1	159.6	±	52.7	158.6	157.8	±	53.9	151.0	-1.8	0.687	-1
Vitamin B_6_ (mg/1000 kcal)	0.8	±	0.1	0.8	0.8	±	0.2	0.8	0.0	0.039	-4	0.8	±	0.1	0.8	0.8	±	0.1	0.8	0.0	0.734	0
Vitamin B_12_ (*µ*g/1000 kcal)	4.9	±	2.5	4.2	4.7	±	2.5	4.3	-0.3	0.229	-6	5.2	±	2.8	4.5	4.9	±	2.1	4.6	-0.3	0.297	-5
Folate (*µ*g/1000 kcal)	136.5	±	36.4	136.0	132.7	±	31.4	131.7	-3.8	0.231	-3	161.1	±	40.1	154.9	161.6	±	41.8	155.3	0.5	0.901	0
Selenium (*µ*g/1000 kcal)	53.7	±	14.8	52.9	52.7	±	16.6	52.7	-1.0	0.531	-2	571	±	13.8	56.6	57.2	±	13.7	57.5	0.0	0.991	0
Total dietary fiber (g/1000 kcal)	6.1	±	1.8	5.7	6.3	±	2.0	6.0	0.2	0.314	3	7.8	±	2.0	7.5	8.0	±	2.2	7.6	0.2	0.243	3
Water-soluble fiber (g/1000 kcal)	1.0	±	0.4	0.9	1.0	±	0.5	0.9	0.0	0.249	5	1.3	±	0.5	1.3	1.4	±	0.5	1.3	0.1	0.206	4
Water-insoluble fiber (g/1000 kcal)	4.3	±	1.3	4.1	4.4	±	1.3	4.1	0.1	0.420	2	5.5	±	1.5	5.2	5.6	±	1.6	5.4	0.1	0.341	2
Daidzein (mg/1000 kcal)	7.5	±	4.1	6.6	8.3	±	5.8	7.5	0.8	0.070	11	8.9	±	4.8	7.7	9.0	±	4.5	8.6	0.1	0.660	2
Genistein (mg/1000 kcal)	12.6	±	6.9	11.3	14.1	±	9.7	12.9	1.4	0.061	11	15.0	±	8.1	13.1	15.6	±	7.9	14.8	0.6	0.294	4

^1^Food frequency questionnaire administered in February, 1995. ^2^Food frequency questionnaire administered in February, 1996

^3^Differences between FFQ1 and FFQ2 were tested by paired t-test. ^4^((FFQ2 mean - FFQ1 mean)/FFQ1 mean)

^5^Saturated fatty acid. ^6^Monounsaturated fatty acid. ^7^Polyunsaturated fatty acid.

Table 3 shows the mean and SD for food intake by food group in crude values and the differences between the two FFQs. The variation in the differences was greater than in nutrient intakes, i.e., the difference was more than 5% in 11 food groups within the 21 food groups examined both in men and women. The variation in the differences in energy-density values was similar to that in crude values (Table 4).

**Table 3.** Means and standard deviations of food group intakes (crude values) assessed with two FFQs administered at one-year interval

Intake	Men (n=101)											Women (n=108)										
	
	FFQ1^1^				FFQ2^2^				Difference	p-value^3^	%^4^ difference	FFQ1^1^				FFQ2^2^				Difference	p-value^3^	%^4^ difference
			
	Mean	±	SD	Median	Mean	±	SD	Median				Mean	±	SD	Median	Mean	±	SD	Median			
Cereals (g/day)	220	±	93	201	241	±	201	200	22	0.300	10	191	±	77	173	202	±	89	186	11	0.144	6
Potatoes and starches (g/day)	30	±	32	22	29	±	29	24	-1	0.748	-2	37	±	43	28	37	±	29	28	-1	0.850	-1
Confectioneries (g/day)	16	±	22	11	18	±	20	13	2	0.452	10	27	±	36	16	26	±	25	18	-1	0.726	-4
Fats and oils (g/day)	13	±	7	13	14	±	9	11	0	0.809	2	14	±	10	11	14	±	7	13	0	0.730	2
Nuts and seeds (g/day)	2	±	3	1	3	±	5	1	1	0.042	37	3	±	8	1	3	±	4	1	0	0.700	9
Pulses (g/day)	69	±	48	62	73	±	55	60	4	0.401	6	64	±	38	54	69	±	39	64	5	0.092	8
Fish and shellfish (g/day)	108	±	81	82	104	±	87	94	-4	0.633	-4	102	±	102	75	94	±	59	81	-8	0.391	-8
Meats (g/day)	68	±	46	57	66	±	53	57	-2	0.724	-3	56	±	40	46	60	±	50	50	4	0.364	7
Eggs (g/day)	31	±	16	25	31	±	24	25	1	0.793	2	30	±	17	25	33	±	28	25	2	0.327	8
Milks (g/day)	183	±	224	130	192	±	222	167	8	0.748	5	195	±	161	200	204	±	227	178	9	0.672	5
Vegetables (g/day)	242	±	169	215	234	±	136	208	-8	0.594	-3	279	±	235	233	278	±	165	238	-1	0.974	0
Green & yellow (g/day)	93	±	88	74	82	±	53	70	-11	0.190	-11	110	±	93	88	110	±	77	88	-1	0.930	-1
Others (g/day)	103	±	64	95	104	±	67	90	1	0.837	1	122	±	123	98	123	±	78	101	1	0.904	1
Pickled (g/day)	46	±	54	24	48	±	54	34	2	0.659	3	47	±	55	25	46	±	48	30	-1	0.806	-2
Fruits (g/day)	208	±	246	153	199	±	165	148	-10	0.573	-5	276	±	318	184	256	±	176	203	-20	0.446	-7
Fungi (g/day)	10	±	9	8	10	±	9	9	0	0.596	5	12	±	9	9	12	±	8	10	0	0.675	3
Algae (g/day)	11	±	8	9	13	±	11	11	2	0.114	17	12	±	8	11	14	±	13	11	2	0.161	13
Beverages (g/day)	1211	±	637	1094	1167	±	569	1133	-44	0.498	-4	817	±	515	688	818	±	415	737	1	0.981	0
Alcoholic beverages (g/day)	303	±	336	180	272	±	309	180	-30	0.314	-10	20	±	72	0	9	±	31	0	-11	0.079	-56
Non-alcohlic beverages (g/day)	909	±	613	720	895	±	533	744	-14	0.799	-2	797	±	514	686	809	±	412	719	12	0.771	1
Seasonings and spices (g/day)	4	±	4	3	4	±	3	3	0	0.473	-6	5	±	5	4	5	±	3	4	0	0.354	-7

^1^Food frequency questionnaire administered in February, 1995. ^2^Food frequency questionnaire administered in February, 1996

^3^Differences between FFQ1 and FFQ2 were tested by paired t-test. ^4^((FFQ2 mean - FFQ1 mean)/FFQ1 mean)

**Table 4.** Means and standard deviations of food group intakes (energy-density values) assessed with two FFQs administered at one year interval

	Men (n=101)											Women (n=108)										
	
	FFQ1^1^				FFQ2^2^				Difference	p-value^3^	%^4^ difference	FFQ1^1^				FFQ2^2^				Difference	p-value^3^	%^4^ difference
			
	Mean	±	SD	Median	Mean	±	SD	Median				Mean	±	SD	Median	Mean	±	SD	Median			
Cereals (g/1000 kcal)	98	±	36	90	103	±	63	89	5	0.399	6	100	±	32	96	100	±	31	96	0	0.987	0
Potatoes and starches (g/1000 kcal)	13	±	13	10	13	±	12	11	0	0.995	0	17	±	14	14	18	±	12	16	0	0.689	2
Confectioneries (g/1000 kcal)	7	±	8	5	8	±	9	5	1	0.181	16	13	±	12	8	12	±	11	9	0	0.865	-1
Fats and oils (g/1000 kcal)	6	±	2	6	6	±	3	5	0	0.700	-1	7	±	3	6	7	±	3	6	0	0.214	4
Nuts and seeds (g/1000 kcal)	1	±	1	1	1	±	2	1	0	0.027	40	1	±	2	1	1	±	2	1	0	0.031	31
Pulses (g/1000 kcal)	29	±	17	26	32	±	23	26	3	0.164	9	33	±	19	28	34	±	18	32	1	0.476	3
Fish and shellfish (g/1000 kcal)	44	±	23	41	43	±	27	41	-1	0.583	-3	47	±	26	42	45	±	21	43	-2	0.383	-5
Meats (g/1000 kcal)	30	±	18	28	28	±	18	25	-2	0.328	-6	27	±	14	25	28	±	17	25	1	0.553	3
Eggs (g/1000 kcal)	14	±	7	13	13	±	9	12	0	0.841	-1	16	±	10	14	16	±	10	14	0	0.954	0
Milks (g/1000 kcal)	75	±	74	65	82	±	93	60	6	0.501	8	102	±	78	93	100	±	105	82	-3	0.764	-3
Vegetables (g/1000 kcal)	103	±	54	93	100	±	48	97	-3	0.487	-3	135	±	67	124	140	±	75	123	5	0.476	4
Green & yellow (g/1000 kcal)	39	±	26	34	35	±	19	31	-4	0.126	-9	54	±	28	49	55	±	34	45	1	0.653	3
Others (g/1000 kcal)	45	±	26	38	45	±	26	40	0	0.954	0	59	±	40	51	63	±	40	49	4	0.347	6
Pickled (g/1000 kcal)	19	±	21	11	20	±	21	12	1	0.631	4	22	±	23	14	22	±	21	14	0	0.852	-1
Fruits (g/1000 kcal)	84	±	72	68	83	±	62	70	-2	0.746	-2	127	±	89	107	126	±	82	109	-1	0.897	-1
Fungi (g/1000 kcal)	4	±	3	3	4	±	3	3	0	0.480	6	6	±	4	5	6	±	4	5	0	0.447	5
Algae (g/1000 kcal)	5	±	3	4	6	±	5	4	1	0.112	17	6	±	4	5	7	±	6	5	1	0.304	9
Beverages (g/1000 kcal)	542	±	284	546	514	±	244	467	-29	0.313	-5	445	±	320	366	426	±	247	393	-19	0.448	-4
Alcoholic beverages (g/1000 kcal)	140	±	170	85	118	±	141	82	-21	0.115	-15	8	±	30	0	4	±	16	0	-4	0.123	-49
Non-alcoholic beverages (g/1000 kcal)	403	±	271	335	395	±	243	325	-7	0.766	-2	437	±	322	353	422	±	245	385	-15	0.554	-3
Seasonings and spices (g/1000 kcal)	2	±	2	1	2	±	1	1	0	0.358	-7	2	±	2	2	2	±	2	2	0	0.427	-5

^1^Food frequency questionnaire administered in February, 1995. ^2^Food frequency questionnaire administered in February, 1996

^3^Differences between FFQ1 and FFQ2 were tested by paired t-test. ^4^((FFQ2 mean - FFQ1 mean)/FFQ1 mean)

Table 5 shows Spearman rank correlation coefficients for nutrients between the two FFQs. The correlation coefficients for crude nutrient intakes varied from 0.41 in vitamin B_12_ to 0.83 in alcohol in men, and from 0.52 in alpha-carotene to 0.77 in iron in women. The median correlation coefficients for crude nutrient intakes were 0.59 and 0.67 in men and women, respectively. After nutrient intakes were adjusted for energy intake, the correlation coefficients varied from 0.30 in vitamin B_12_ to 0.82 in alcohol in men, and from 0.32 in protein to 0.68 in alcohol in women. The median correlation coefficients for energy-adjusted nutrient intakes were 0.49 and 0.50 in men and women, respectively.

**Table 5.** Spearman rank correlation coefficients between two FFQs administered at one-year interval for estimated nutrient intakes

Energy and Nutrients	Men (n=101)		Women (n=108)	
	
	Crude	Energy-adjusted^1^	Crude	Energy-adjusted^1^
Energy	0.52	---	0.67	---
Protein	0.59	0.47	0.69	0.32
Total fat	0.53	0.47	0.67	0.52
SFA^2^	0.53	0.53	0.61	0.51
MUFA^3^	0.53	0.51	0.67	0.60
PUFA^4^	0.59	0.40	0.73	0.46
n-3 PUFA^4^	0.61	0.51	0.74	0.41
n-6 PUFA^4^	0.59	0.37	0.72	0.49
Carbohydrate	0.66	0.45	0.66	0.50
Alcohol	0.83	0.82	0.67	0.68
Calcium	0.63	0.49	0.67	0.56
Phosphorus	0.63	0.47	0.67	0.49
Iron	0.64	0.49	0.77	0.50
Sodium	0.64	0.49	0.73	0.63
Potassium	0.57	0.45	0.65	0.49
Retinol	0.49	0.52	0.56	0.41
Carotene	0.51	0.40	0.56	0.46
Alpha-carotene	0.50	0.48	0.52	0.42
Beta-carotene	0.53	0.43	0.56	0.46
Vitamin B_1_	0.51	0.43	0.56	0.54
Vitamin B_2_	0.59	0.43	0.69	0.52
Niacin	0.55	0.43	0.64	0.35
Vitamin C	0.70	0.67	0.58	0.49
Cholesterol	0.56	0.51	0.69	0.54
Vitamin B_6_	0.58	0.43	0.68	0.52
Vitamin B_12_	0.41	0.30	0.66	0.38
Folate	0.57	0.45	0.69	0.53
Selenium	0.55	0.51	0.60	0.48
Total dietary fiber	0.66	0.54	0.63	0.48
Water-soluble fiber	0.70	0.61	0.65	0.53
Water-insoluble fiber	0.67	0.52	0.64	0.46
Daidzein	0.75	0.66	0.76	0.66
Genistein	0.72	0.60	0.75	0.61
Median	0.59	0.49	0.67	0.50

^1^Nutrient intakes were adjusted for energy intake by residual method.

^2^Saturated fatty acid. ^3^Monounsaturated fatty acid. ^4^Polyunsaturated fatty acid.

Table 6 shows Spearman rank correlation coefficients for food groups between the two FFQs. The correlation coefficients for crude food intake by food group varied from 0.42 in seasonings and spices to 0.80 in pickled vegetables in men, and from 0.45 in seasonings and spices to 0.74 in pulses, milks, and pickled vegetables in women. The median correlation coefficients for crude food intakes were 0.61 and 0.63 in men and women, respectively. After food intakes by food groups were adjusted for energy intake, the correlation coefficients varied from 0.38 in total beverages to 0.71 in alcoholic beverages in men, and from 0.30 in nuts and seeds to 0.74 in milks in women. The median correlation coefficients for energy-adjusted food intakes were 0.50 and 0.49 in men and women, respectively.

**Table 6.** Spearman rank correlation coefficients between two FFQs administered at one-year interval for estimated nutrient intakes

Food groups	Men (n=101)		Women (n=108)	
	
	Crude	Energy-adjusted^1^	Crude	Energy-adjusted^1^
Cereals	0.43	0.43	0.53	0.37
Potatoes and starches	0.56	0.53	0.63	0.48
Confectioneries	0.71	0.63	0.71	0.36
Fats and oils	0.55	0.67	0.69	0.51
Nuts and seeds	0.73	0.43	0.61	0.30
Pulses	0.76	0.64	0.74	0.67
Fish and shellfish	0.65	0.44	0.61	0.34
Meats	0.53	0.52	0.55	0.52
Eggs	0.52	0.49	0.66	0.46
Milks	0.64	0.48	0.74	0.74
Vegetables	0.67	0.62	0.63	0.53
Green & yellow	0.51	0.47	0.53	0.49
Others	0.61	0.62	0.59	0.46
Pickled	0.80	0.66	0.74	0.68
Fruits	0.72	0.50	0.67	0.50
Fungi	0.58	0.46	0.54	0.48
Algae	0.48	0.41	0.56	0.44
Beverages	0.46	0.38	0.65	0.59
Alcoholic beverages	0.71	0.71	0.66	0.67
Non-alcoholic beverages	0.69	0.55	0.63	0.58
Seasonings and spices	0.42	0.41	0.45	0.40
Median	0.61	0.50	0.63	0.49

^1^Food intakes were adjusted for energy intake by residual method.

## DISCUSSION

In the present study, we examined the reproducibility of the FFQ which was repeatedly administered at a one-year interval for estimating dietary intake of nutrients and foods. We compared two repeated measurements by Spearman rank correlation coefficients, which ranged from 0.41 to 0.83 among various nutrients for crude nutrient intake in men and women. The correlation coefficients for most of the nutrients were higher in women than in men, but lower after energy adjustment both in men and women. The correlation was relatively high in nutrients, such as n-3 polyunsaturated fatty acid, vitamin C, daidzein and genistein, whose food sources were limited to a few food items. The correlation coefficients for crude food intake ranged from 0.42 to 0.80 among various food groups in men and women. Correlation coefficients for men and women were similar, and lower after energy adjustment; they were relatively high for confectionaries, pickled vegetables, and alcoholic beverages which tended to be consumed according to individual preferences.

Reproducibility of our FFQ was comparable to the results from the validation study of similar questionnaires previously developed in Japan. Correlation coefficients between repeated measurements in our study for selected nutrients were compared to that in two other similar questionnaires in Table 7.^6^^-^^8^ Our results indicated the same declining tendency as the study by Imaeda et al.^8^ after nutrient intakes were adjusted for energy. The correlation coefficients also decreased for food groups after the energy adjustment in our study. The same tendency after energy adjustment was observed in the validity of our questionnaire in estimating nutrients and food groups.^9^^,^^10^ Correlation coefficients were slightly higher in women than in men for most of the nutrient and food groups, in contrast to where the validity was concerned.^9^^,^^10^

**Table 7.** Comparison of reproducibility of self-administered food frequency questionnaires developed for epidemiologic studies in Japan: results expressed by correlation coefficients for energy and 8 selected nutrients

Study	Shimizu et al.		Egami et al.	Imaeda et al.	Present study	
Type of correlation	IC	IC	PC	SC	SC	SC
Sex	Men	Women	Both	Women	Men	Women
n	58	59	86	84	101	108
Crude intakes						
Energy	0.53	0.30	0.58	0.78	0.52	0.67
Protein	0.54	0.33	0.52	0.74	0.59	0.69
Fat	0.65	0.45	0.58	0.71	0.53	0.67
Carbohydrate	0.45	0.24	0.58	0.73	0.66	0.66
Calcium	0.67	0.50	0.76	0.73	0.63	0.67
Carotene	0.49	0.60	0.53	0.62	0.64	0.73
Vitamin C	0.51	0.18	0.66	0.65	0.51	0.56
Sodium	0.55	0.32	---	---	0.70	0.58
Cholesterol	0.67	0.48	0.41	0.80	0.56	0.69
Median	0.54	0.33	0.58	0.73	0.59	0.67
Energy-adjusted intakes*						
Protein	0.52	0.50	0.65	0.56	0.47	0.32
Fat	0.61	0.57	0.74	0.52	0.47	0.52
Carbohydrate	0.65	0.49	0.53	0.51	0.45	0.50
Calcium	0.78	0.67	0.82	0.70	0.49	0.56
Carotene	0.62	0.58	0.48	0.58	0.49	0.63
Vitamin C	0.64	0.13	0.68	0.56	0.40	0.46
Sodium	0.55	0.48	---	---	0.67	0.49
Cholesterol	0.55	0.36	0.51	0.46	0.51	0.54
Median	0.62	0.50	0.65	0.56	0.48	0.51

Abbreviations: IC=intraclass correlation; PC=Pearson correlation; SC=Spearman correlation.

*Residual method

Correlation coefficients between repeated measurements for estimating dietary intake are usually on the order of 0.5-0.7.^1^ This level of reproducibility is comparable to that of many biological measurements such as serum cholesterol and blood pressure, which are strong and consistent predictors of disease in epidemiologic studies.^1^ Except for vitamin B_12_, cereals, algae, and seasoning and spices, the reproducibility of our FFQ for estimating intake of nutrients and food groups was r>0.5, which was sufficiently reasonable for epidemiologic use. With the reasonable validity of our FFQ,^9^^,^^10^ we can be sure that our FFQ at one point in time can determine the usual intakes of individuals over the period of one year.

In conclusion, the reproducibility of the FFQ used in the JPHC Study cohort I in estimating most of the nutrient and food group intakes was comparable to that of the other questionnaires developed in Japan.
